# The dynamics of pico-sized and bloom-forming cyanobacteria in large water bodies in the Mekong River Basin

**DOI:** 10.1371/journal.pone.0189609

**Published:** 2017-12-22

**Authors:** Michio Fukushima, Noriko Tomioka, Tuantong Jutagate, Mikiya Hiroki, Tomoyoshi Murata, Chatchai Preecha, Piyathap Avakul, Pisit Phomikong, Akio Imai

**Affiliations:** 1 Center for Environmental Biology and Ecosystem Studies, National Institute for Environmental Studies, Tsukuba, Japan; 2 Center for Regional Environmental Research, National Institute for Environmental Studies, Tsukuba, Japan; 3 Faculty of Agriculture, Ubon Ratchathani University, Ubon Ratchathani, Thailand; 4 Faculty of Applied Science and Engineering, Khon Kaen University, Nong Khai, Thailand; 5 Mahidol University, Nakhon Sawan Campus, Nakhon Sawan, Thailand; 6 Inland Fisheries Research and Development Division, Department of Fisheries, Bangkok, Thailand; 7 Lake Biwa Branch Office, National Institute for Environmental Studies, Otsu, Japan; University of Hyogo, JAPAN

## Abstract

In the face of plans for increased construction of dams and reservoirs in the Mekong River Basin, it is critically important to better understand the primary-producer community of phytoplankton, especially the warm-water cyanobacteria. This is because these algae can serve as the primary source of carbon for higher trophic levels, including fishes, but can also form harmful blooms, threatening local fisheries and environmental and human health. We monitored the dynamics of three cyanobacteria—*Synechococcus* spp., *Microcystis aeruginosa*, and *Dolichospermum* spp.—for two years in nine large lakes and reservoirs in the Mekong River Basin. The densities of these algae were largely system-specific such that their abundance was uniquely determined within individual water bodies. However, after accounting for the system-specific effect, we found that cell densities of *Synechococcus* spp., *M*. *aeruginosa*, and *Dolichospermum* spp. varied in response to changes in photosynthetically active radiation (PAR), total nitrogen, and water level, respectively. Because both PAR and water level tend to fluctuate concordantly over a wide geographic area, *Synechococcus* spp., and to a lesser extent *Dolichospermum* spp., varied synchronously among the water bodies. Sustaining the production of pico-sized primary producers while preventing harmful algal blooms will be a key management goal for the proposed reservoirs in the Mekong Basin.

## Introduction

The Mekong River, an international river flowing through six countries on the Indochina Peninsula of Southeast Asia, has been dammed primarily for hydropower generation and irrigation since the 1960s. Increasing demand for electricity has prompted nearly 100 proposals for additional dams throughout the basin [1]. However, hydropower development along the Mekong is highly controversial because of the world’s largest inland fishery [2] that provides local residents with a vital source of animal protein [3].

The enormous fish production in the Mekong is undoubtedly attributable to massive primary production by algae and plants in the river system. Despite significant concerns over damming, one can argue that overall algal production might be enhanced if numerous dams and the resultant shallow water bodies (i.e., reservoirs) are constructed throughout the basin. Furthermore, reservoir fisheries may compensate for the loss of traditional capture fisheries if the waters are properly managed for aquaculture [4]. To determine the validity of this argument, it is critically important to better understand the dynamics of primary producers in the existing water bodies of the Mekong River Basin.

The key to successful management of existing and future water bodies for sustained, productive fisheries may lie in the elucidation of the ecological roles of small (0.2–2 μm) picophytoplankton, especially those of its major taxonomical component, the picocyanobacteria [5,6]. Among aquatic ecologists, there is increasing interest in picocyanobacteria because they often become predominant not only in oligo–mesotrophic waters [7,8] but also in hypertrophic shallow waters under certain conditions, as discussed later [9], contributing substantially to total primary production regardless of trophic status [10,11]. They seldom create harmful blooms [12], are readily available to microorganisms such as heterotrophic nanoflagellates and small ciliates [13,14], and are therefore a very important food-web component in aquatic systems [5,6,14]. Despite relatively extensive literature on these smallest autotrophs, their dynamics, distribution, and limiting factors are less well understood for tropical, freshwater systems compared to temperate, saltwater systems [6,8,15].

If excess algal production develops into harmful blooms, it will pose a serious threat not only to fisheries but also to environmental and human health [16,17]. *Microcystis* spp. and their toxin (i.e., microcystin) have already been found in drinking water from watersheds supporting big cities and popular tourist destinations in Southeast Asia [18–20]. Recent marked economic growth in the Mekong riparian countries has promoted rapid population growth in urban areas and boosted agricultural production in rural areas [21]. As a result, nutrient loading to the Mekong River has risen considerably through increased wastewater discharge and extensive fertilizer use [22], possibly accelerating eutrophication in the existing waters. These water bodies commonly are shallow, receive substantial solar radiation, and hold warm water with a relatively long residence time due to flat topography [23,24]. Therefore, even aside from the recent nutrient enrichment of the river system, the risk of harmful algal blooms is inherently high in the region [20]. Nevertheless, there have been no specific measures proposed to tackle or to prevent algal blooms for the existing or future water bodies [25].

We investigated the dynamics of three coexisting, dominant cyanobacteria in the Mekong River Basin, namely *Synechococcus* spp., *Microcystis aeruginosa*, and *Dolichospermum* spp., by monitoring multiple large water bodies of various trophic status for two years. Our primary objective was to predict the occurrence and abundance of both pico-sized and bloom-forming cyanobacteria in the existing water bodies and to infer the same for proposed future reservoirs. The dynamics of picocyanobacteria have neither been investigated in the Mekong River Basin, nor, to our knowledge, been examined in conjunction with those of coexisting large bloom-forming cyanobacteria in any freshwater system using the same methodology.

## Methods

### Ethics statement

Permission was obtained from the Living Aquatic Resources Research Center of the Ministry of Agriculture and Forestry, Lao PDR, from the Inland Fisheries Research and Development Bureau, Department of Fisheries, Thailand, and from the Inland Fisheries Research and Development Institute, Cambodia, for sampling in lakes and reservoirs in the respective countries. Sampling was not conducted in protected areas and did not involve any endangered or protected species.

### Study area

**Fig 1:**
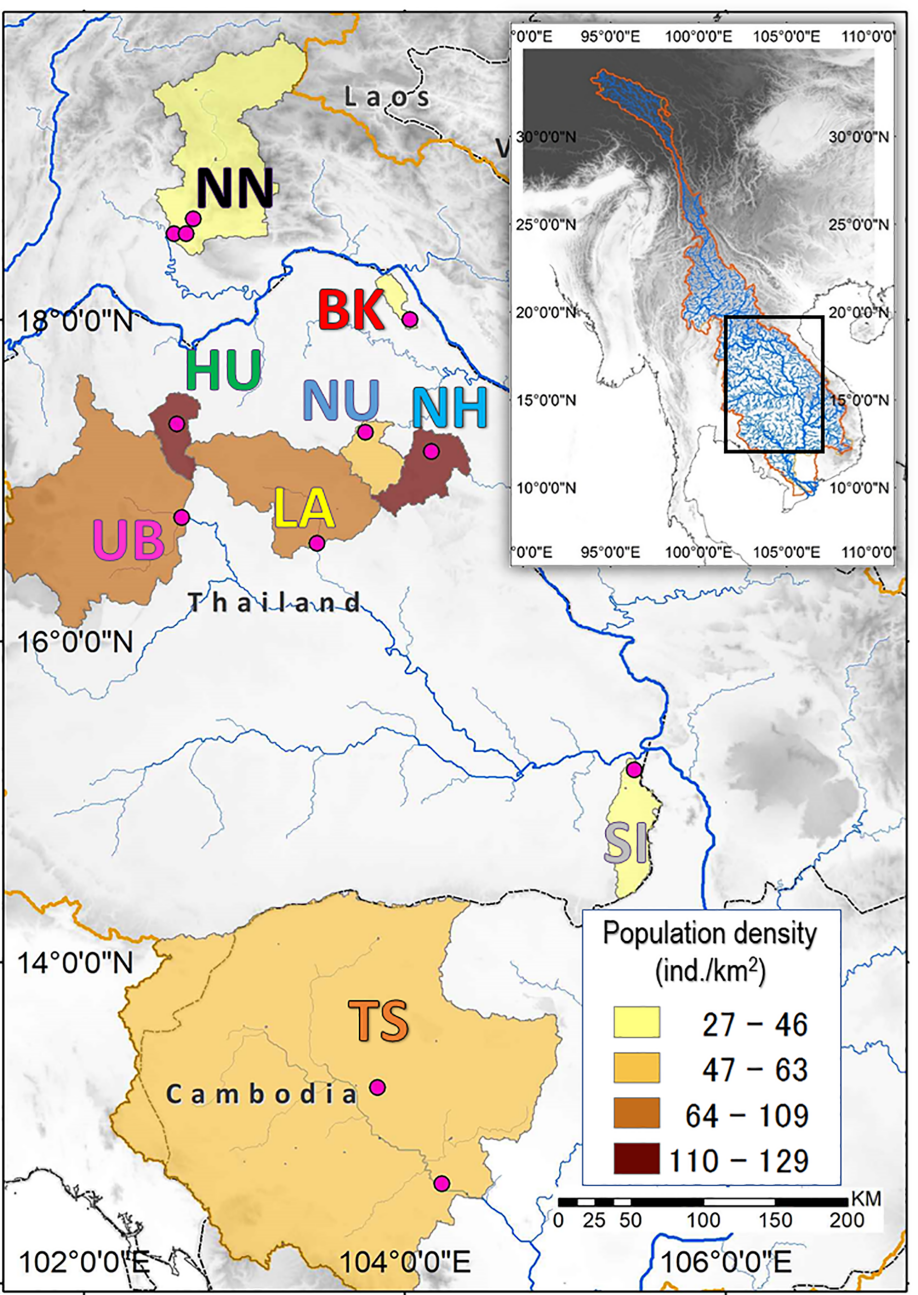
Map of the study area.

Nine large water bodies comprising three lakes and six reservoirs were periodically sampled for two years at 3- to 4-month intervals during 2012–2014. All water bodies are located between latitudes 12°N and 19°N in the Mekong River Basin of Lao PDR, Thailand, and Cambodia (Fig 1; Table 1 where climate data are from the CRU TS 2.1 dataset [26]). Sampling was repeated five to eight times in most water bodies; three were visited only three times during 2013–2014: Bueng Khong Long (BK), Nam Un (NU), and Nong Han (NH). A sampling station was established in each water body around the deepest area, close to a dam in the case of reservoirs. The two largest water bodies, Nam Ngum (NN) and Tonle Sap (TS), were sampled at three and two stations, respectively; all of the measurements including algal densities were averaged to represent each water body for statistical analysis.

**Table 1:** Characteristics of water bodies investigated in this study.

Water body	Altitude (m)	Mean depth (m)	Surface area (km^2^)	Catchment area (km^2^)	Mean air temp. (°C)	Mean precipitation (mm/month)	Fish production^a^ (kg/ha)
Nam Ngum (NN)	208	18.9	370	8297	24.7	90	40–185
Bueng Khong Long (BK)	160	0.8	22	412	27.4	79	-
Huay Luang (HU)	198	3.6	31	1186	27.9	77	252
Nam Un (NU)	179	6.4	86	1288	27.7	76	25/121
Nong Han (NH)	155	1.9	135	1898	27.7	75	26
Ubolratana (UB)	179	15.8	410	12,116	27.8	79	55/61
Lam Pao (LA)	160	5.5	230	6057	27.9	76	66
Sirindhorn (SI)	141	5.1	292	2094	27.6	77	44
Tonle Sap (TS)	4	3–7	2500–17,500	54,986	27.9	124	230

^a^Two independent estimates of annual fish production are shown for NU and UB, whereas no estimate is available for BK.

Data source other than climate data: Hortle [23] and Jutagate [24].

The watersheds of the nine water bodies studied are shaded in proportion to population density (ind./km^2^) [27]. Sampling stations are denoted by pink dots. River network and elevation data are based on HydroSHED [28], and shoreline data were obtained from the Global Self-consistent, Hierarchical, High-resolution Geography Database (GSHHG) [29].

Annual mean air temperature reaches 27–28°C at all bodies of water except for NN (<25°C) (Table 1). Average rainfall is extremely high for TS (124 mm/month), followed by NN (90 mm/month), with the remaining water bodies receiving substantially less rainfall (75–79 mm/month) as they are located on the semi-arid Korat Plateau of Thailand [30]. TS drastically changes in water depth (3–7 m) and surface area (2500–17,500 km^2^) in an annual monsoon cycle of dry and wet seasons. Average annual fish production per unit area is highest in the smallest water body, Huay Luang (HU, 252 kg/ha), followed by the largest water body TS (230 kg/ha). Although oligotrophic, as described later, both NN and Sirindhorn (SI) have relatively high annual fish yields (NN, 40–185 kg/ha; SI, 44 kg/ha). The watersheds of HU, NH, and Lam Pao (LA) are densely populated (>100 individuals/km^2^) compared to those of NN, BK, and SI (<50 individuals/km^2^) (Fig 1). Although BK and NH are natural lakes, they were artificially impounded in 1979 and 1953, respectively, to expand their water storage capacities for irrigation [31].

### Physical measurements

Water temperature, dissolved oxygen, and pH were measured with a multiparameter mini-sonde (Hydrolab MS5; OTT Hydromet, Inc., Loveland, CO, USA) at the water surface at each sampling station. Secchi-disk transparency was also measured. Illuminance was recorded at 1-h intervals during the entire study period by multiple data loggers (HOBO UA-002-64; Onset, Bourne, MA, USA) deployed on the roofs of university buildings in the three countries. Photosynthetically active radiation (PAR) was then estimated for each water body during the same period based on linear regression between in situ PAR measurements during field sampling using an underwater quantum sensor (LI-192SA; Li-Cor, Inc., Lincoln, NE, USA) and the corresponding illuminance data from the nearest data logger. The PAR estimates during the month prior to each sampling were then averaged to represent light available to algae for that sampling in each water body.

### Nutrient analysis

One liter of surface water was collected from a boat at the sampling station in each water body. For NN and TS, water was collected from all three and both sampling stations, respectively. A 100-mL subsample of the water was filtered through a Whatman GF/F filter within 3 h of sampling for nutrient analysis. The filtrate was acidified with hydrochloric acid to 0.02 mol/L and refrigerated. For analysis, the filtrate was neutralized with sodium hydroxide and concentrations of orthophosphate (PO_4_-P), ammonium (NH_4_^+^), nitrate (NO_3_^–^), and nitrite (NO_2_^–^) were determined by using a continuous flow autoanalyzer (QuAAtro2-HR; BL TEC K.K., Osaka, Japan). Dissolved inorganic nitrogen (DIN) was defined as the sum of NO_2_-N, NO_3_-N, and NH_4_-N. Dissolved total nitrogen (DTN) and phosphorus (DTP) were determined with the alkaline persulfate digestion technique [32].

Immediately after sampling, formaldehyde was added to an unfiltered water sample (final concentration of 1%) to terminate photosynthesis; this sample was later analyzed for dissolved inorganic carbon (DIC) concentration using a total organic carbon (TOC) analyzer (TOC-V_CPH_; Shimadzu Corp., Kyoto, Japan) [33]. Total suspended solids (SS) were measured by filtering the water through a pre-weighed GF/F filter that was then dried at 105°C overnight. The same filter was subsequently analyzed for particulate organic nitrogen (PON) using an elemental analyzer (JM-10; J-Science Lab, Osaka, Japan). Total nitrogen (TN) was obtained as the sum of DTN and PON, whereas total phosphorus (TP) was obtained by using the same method as for DTP except that unfiltered water was analyzed instead of filtrate. Chlorophyll *a* (chl *a*) concentration was determined spectrophotometrically (UV-2550; Shimadzu Corp.) after methanol extraction of particles collected from the water sample on a GF/F filter.

### Extraction of cyanobacterial DNA

Depending on the turbidity, 100–150 mL of surface water was filtered through a 47-mm Whatman GF/F filter to collect an algal sample for DNA extraction. The filter was immediately sprayed with 75% ethanol to preserve DNA, wrapped in aluminum foil, and refrigerated during transportation to the laboratory, where it was stored at –80°C until extraction. DNA was extracted from microorganisms using the Extrap Soil DNA Kit Plus ver. 2 (Nippon Steel & Sumikin Eco-Tech Corp., Tokyo, Japan) according to the manufacturer’s instructions. In brief, each filter was cut into small pieces and placed in a bead-beating tube, to which 950 μL of extraction buffer and 50 μL of lysis solution were added. Algal cells were disrupted using a homogenizer (FastPrep; MP Biomedicals, LLC, Santa Ana, CA, USA) at a speed of 6.0 m/s for 40 s, after which DNA was purified by using magnetic beads to produce 100 μL of DNA extract.

### Real-time quantitative PCR

Real-time quantitative polymerase chain reaction (qPCR) was performed using specific primers, some of which were designed as part of this study based on nucleotide sequences available from DDBJ/EMBL/GenBank databases (Table 2). Because there is high 16S rDNA identity between the genera *Dolichospermum* and *Aphanizomenon* [34,35], we carefully examined primer specificity to the former and ascertained that 11 of 14 *Dolichospermum* strains and one of eight *Aphanizomenon* strains studied by Li et al. [35] were amplified using our primers for *Dolichospermum* spp.

**Table 2:** Primers used for qPCR to amplify 16S rDNA of total cyanobacteria, *Synechococcus* spp., *Microcystis aeruginosa*, and *Dolichospermum* spp.

Target	Primer^a^	Primer sequence (5′–3′)	Amplicon size (bp)	Reference
Cyanobacteria	CYA361F CYA457R	GGAATTTTCCGCAATGGG ACGGAGTTAGCCGTGGCTTATTC	135	Schönhuber et al. [36] This study
*Synechococcus* spp.	Synecho430F Synecho539R	TGAAGGCCTCTGGGCTGTA CGGATAACGCTTGCCACTC	125	This study
*Microcystis aeruginosa*	Micro233F Cyano342R	CTAATTGGCCTGRAGAAGAGC GCTGCCTCCCGTAGGAGT	145	Tomioka et al. [37]
*Dolichospermum* spp.	Dolicho664F Dolicho735R	CTACAAAGCTAGAGTTTGGTCG GTCTCGGCCTAGCAGAACG	109	This study

^a^F, forward primer; R, reverse primer

The real-time qPCR reaction mixture of 20 μL contained LightCycler 480 SYBR Green I Master (Roche, Mannheim, Germany), 10 pmol of each primer, and 1 μL of the DNA extract. Denaturation was performed for 5 min at 95°C, followed by 40 cycles of repeated denaturation (10 s at 95°C), annealing (10 s at 60°C) with fluorescence acquisition (wavelength, 530 nm), and extension (10 s at 72°C). The temperature transition rate was 4.4°C/s for denaturation and extension and 2.2°C/s for annealing. The standards for qPCR were the PCR products of respective cyanobacterial strains (i.e., NIES78, NIES978, NIES843, and NIES78 for total cyanobacteria, *Synechococcus* spp., *M*. *aeruginosa*, and *Dolichospermum* spp., respectively). The copy number of each standard was calculated according to the molecular weight and the density of the PCR product, the latter of which was estimated using Qubit dsDNA HS Assay Kit (Invitrogen, Carlsbad, CA, USA). A standard curve of dilutions of the PCR product was created with each analytical run to serve as a reference for copy numbers of cyanobacterial 16S rDNA. After qPCR, a melting curve analysis was performed by continuous measurement of fluorescence while heating from 65 to 97°C at a transition rate of 0.11°C/s. Each DNA extract sample was assayed twice, and the mean 16S rDNA concentration was used for subsequent analyses.

To determine the relationship between rDNA copy number and biovolume, we used samples of cultured algae (i.e., *Synechococcus* sp. NIES947, *M*. *aeruginosa* NIES843, *Dolichospermum planctonicum* NIES815). Algal cells were collected onto a membrane filter (pore size, 0.2 μm), counted, and measured for approximate dimensions under an epifluorescence microscope (BX53, Olympus, Tokyo, Japan). The same sample was subsequently analyzed for 16S rDNA copy number by qPCR as described above, and the conversion factor between rDNA copy number and biovolume was derived for each alga.

### Statistical analysis

We used principal components analysis to characterize water bodies using the physicochemical variables. Kendall’s coefficients of concordance (*W*) were first calculated to assess overall independence of water bodies with regard to the seasonal patterns of algal densities during the study period [38]. If this statistic was significant, then partial concordance (*W*_*j*_) was calculated for each water body to identify concordant water bodies. The significance of *W* was tested using 9999 permutations of the time series of algal densities in all water bodies, whereas that of *W*_*j*_ was tested using 9999 permutations of the time series of the water body under consideration.

We used simple linear regression to explain the variation of chl *a* concentration against rDNA copy number of total cyanobacteria and the combined biovolume of all three cyanobacteria to assess the contribution of cyanobacteria in general, and the three taxa in particular, to total phytoplankton biomass. Densities of *Synechococcus* spp., *M*. *aeruginosa*, and *Dolichospermum* spp. were modelled separately with multiple linear regression using a stepwise variable selection procedure. Candidates for predictor variables included a categorical variable (water) to represent individual water bodies, sampling season (wet season, from May to October, or dry season, from November to April), physical variables (water depth, temperature, transparency, and PAR), and nutrient concentrations (PO_4_-P, DIN, DIC, DTP, DTN, DTN:DTP, SS, TN, and TP). Algal densities and nutrient concentrations below detection limits were replaced with a value equal to half the corresponding detection limit. Nutrient and chl *a* concentrations and algal densities were logarithmically transformed before regression analysis. A significance level of α = 0.05 was used for all statistical analyses.

## Results

During the study period, the water bodies were 28.0–30.7°C, mildly alkaline (pH = 7.3–9.1), well oxygenated (DO > 6.8 mg/L), and low in suspended solids, except for TS, where the average SS was nearly two orders of magnitude greater than in the others (Table 3). Phytoplankton was most abundant in TS (chl *a* = 20.0 μg/L) and least in NN or NH (1.6 μg/L).

**Table 3:** Environmental parameters in the water bodies (mean ± SD) during the study period as measured at the sampling stations. Water body abbreviations are given in Table 1.

Water body	Water temp. (°C)	pH	DO (mg/L)	SS (mg/L)	PAR (μmol/m^2^/s)	Chl *a* (μg/L)	Secchi depth (m)
NN	28.0 ± 2.2	8.0 ± 0.4	7.9 ± 0.9	1.9 ± 0.5	332 ± 19	1.6 ± 0.9	3.94 ± 0.51
BK	29.3 ± 1.6	7.4 ± 0.5	6.8 ± 0.5	2.6 ± 2.1	297 ± 44	3.3 ± 1.4	2.22 ± 0.93
HU	30.7 ± 2.3	8.2 ± 0.8	8.2 ± 1.3	7.8 ± 4.2	338 ± 25	17.8 ± 10.1	1.16 ± 0.34
NU	30.0 ± 1.7	8.8 ± 0.9	8.0 ± 0.4	3.0 ± 1.5	297 ± 17	14.6 ± 12.2	2.63 ± 0.47
NH	29.4 ± 1.0	9.1 ± 0.5	7.8 ± 0.4	1.2 ± 0.2	320 ± 4	1.6 ± 1.1	2.63 ± 0.69
UB	28.6 ± 1.9	8.2 ± 0.7	7.1 ± 2.1	3.4 ± 1.2	313 ± 45	8.0 ± 2.9	2.10 ± 0.38
LA	28.7 ± 1.8	7.9 ± 0.6	7.4 ± 1.5	4.7 ± 2.5	326 ± 37	6.4 ± 2.4	1.78 ± 0.55
SI	29.3 ± 1.8	7.7 ± 0.5	7.3 ± 1.0	2.2 ± 1.7	307 ± 47	2.8 ± 1.2	3.99 ± 0.95
TS	29.4 ± 1.9	7.3 ± 0.8	8.3 ± 2.2	163 ± 223	308 ± 19	20.0 ± 16.6	0.57 ± 0.56

DO, dissolved oxygen; SS, suspended solids; PAR, photosynthetically active radiation; Chl *a*, chlorophyll *a*.

PO_4_-P was low and relatively constant throughout the study period in all water bodies (<0.008 mg/L, Table 4). Based on the TP levels, NN, BK, NH, and SI were categorized as oligotrophic (<0.01 mg/L), NU, Ubolratana (UB), and LA as mesotrophic (0.01–0.035 mg/L), and HU and TS as eutrophic (>0.035 mg/L). Total nitrogen (TN) ranged from 0.21 to 1.10 mg/L, DIC from 0.43 to 15.10 mg/L, and TN/TP ratios from 34.3 to 157.

**Table 4:** Mean (SD) nutrient concentrations at the sampling stations in water bodies during the study period. Water body abbreviations are given in Table 1.

Water body	PO_4_-P (mg/L)	DTP (mg/L)	TP (mg/L)	DIN (mg/L)	DTN (mg/L)	TN (mg/L)	DIC (mg/L)	TN/TP (mol/mol)
NN	0.0005 (0.0008)	0.002 (0.001)	0.003 (0.002)	0.020 (0.019)	0.109 (0.085)	0.206 (0.091)	8.62 (0.85)	156.5 (125.5)
BK	0.0008 (0.0004)	0.004 (0.001)	0.007 (0.004)	0.012 (0.007)	0.171 (0.061)	0.315 (0.127)	0.43 (0.10)	112.0 (36.6)
HU	0.0025 (0.0018)	0.009 (0.002)	0.041 (0.013)	0.028 (0.039)	0.372 (0.118)	0.774 (0.207)	7.81 (2.74)	44.5 (13.5)
NU	0.0012 (0.0007)	0.007 (0.001)	0.014 (0.007)	0.016 (0.013)	0.288 (0.020)	0.469 (0.068)	2.74 (1.13)	83.5 (23.0)
NH	0.0007 (0.0006)	0.004 (0.002)	0.007 (0.002)	0.009 (0.006)	0.213 (0.022)	0.313 (0.019)	4.23 (1.72)	103.2 (22.0)
UB	0.0014 (0.0019)	0.003 (0.002)	0.010 (0.003)	0.023 (0.021)	0.231 (0.052)	0.411 (0.079)	15.10 (2.11)	91.4 (21.1)
LA	0.0038 (0.0047)	0.006 (0.003)	0.019 (0.003)	0.058 (0.063)	0.306 (0.095)	0.467 (0.097)	5.05 (0.87)	56.3 (10.7)
SI	0.0011 (0.0008)	0.003 (0.001)	0.006 (0.004)	0.024 (0.036)	0.163 (0.023)	0.269 (0.041)	0.49 (0.34)	122.7 (50.4)
TS	0.0074 (0.0050)	0.020 (0.008)	0.069 (0.033)	0.119 (0.102)	0.351 (0.172)	1.104 (0.836)	7.49 (2.06)	34.3 (14.9)

DTP, dissolved total phosphorus; TP, total phosphorus; DIN, dissolved inorganic nitrogen; DTN, dissolved total nitrogen; TN, total nitrogen; DIC, dissolved inorganic carbon.

**Fig 2:**
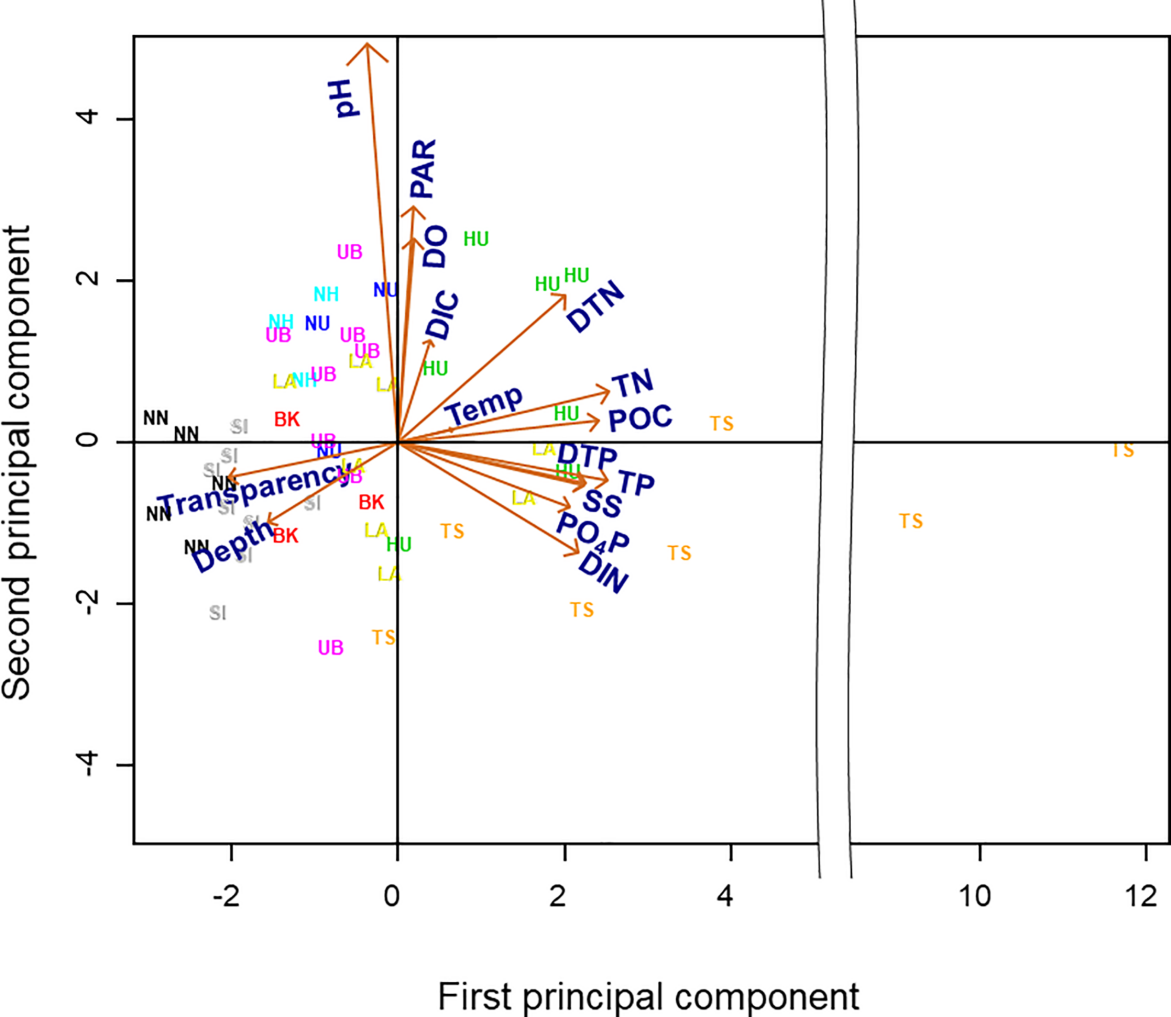
Principal components analysis. Bi-plot of physicochemical variables measured in all water bodies during the entire study period. Water body abbreviations are given in Table 1.

The variability of the environmental parameters was explained reasonably well (56% of variance) by the first and second principal components (PCs) (Fig 2). The nine water bodies were distinctly separated along the first PC, from deep, clear, oligotrophic waters (e.g., NN and SI) to shallow, turbid, eutrophic waters (e.g., HU and TS). In contrast, the second PC separated sampling events within water bodies from low to high photosynthetic activity in terms of PAR, pH, and DO.

Chl *a* concentration across all sampling events and water bodies (*n* = 53) was positively correlated with both total cyanobacterial rDNA copy number (*R*^2^ = 0.37, F_1,51_ = 29.66, *P* < 0.0001) and the combined biovolume of *Synechococcus* spp., *M*. *aeruginosa*, and *Dolichospermum* spp. (*R*^2^ = 0.36, F_1,51_ = 28.48, *P* < 0.0001). This indicates an important contribution from cyanobacteria, especially these three taxa, to phytoplankton communities in the water bodies.

**Fig 3:**
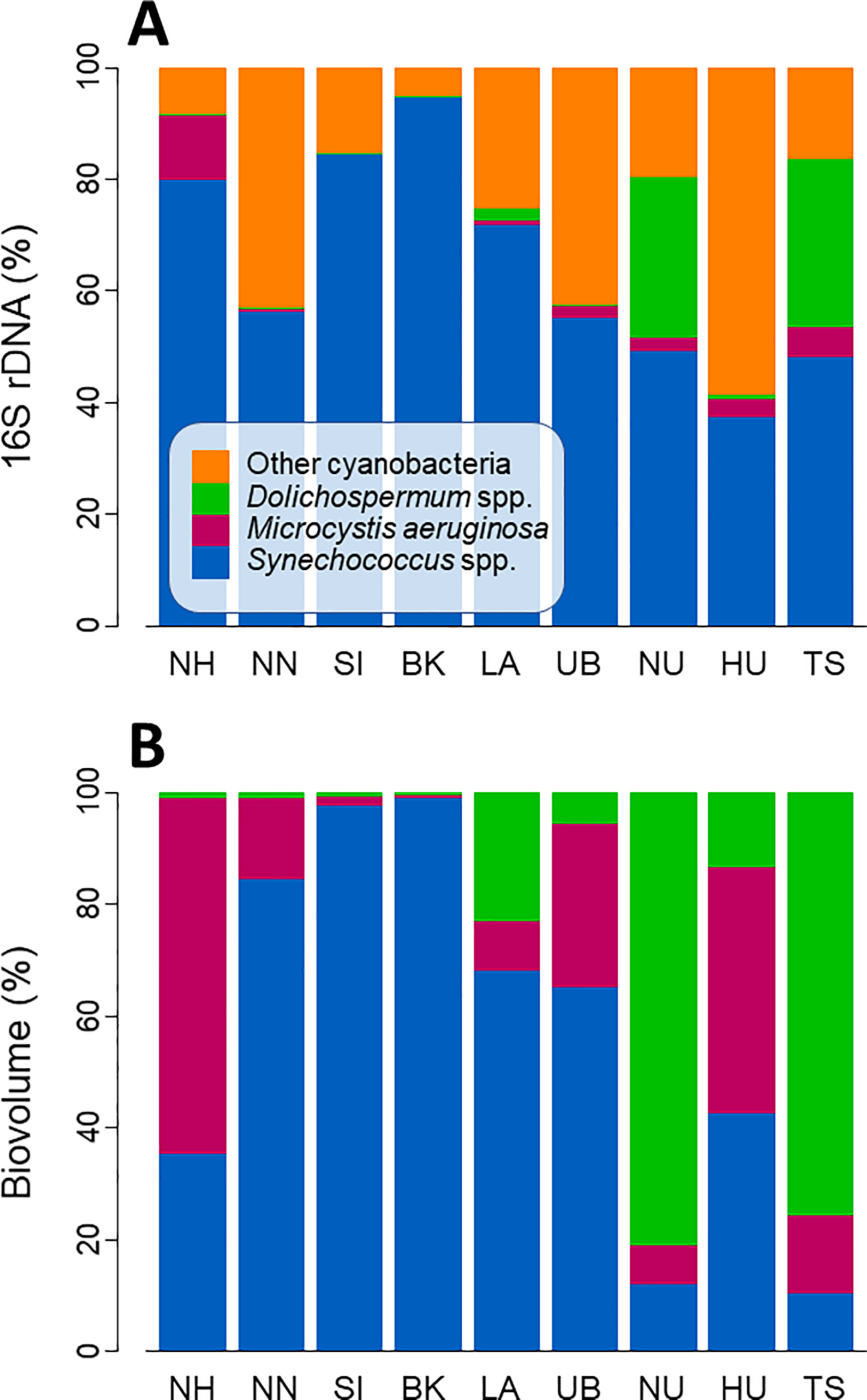
Cyanobacterial composition. Percent composition by (A) rDNA copy numbers for *Synechococcus* spp., *Microcystis aeruginosa*, *Dolichospermum* spp., and other cyanobacteria, and (B) biovolume for the three identified algal taxa. Water bodies are listed from left to right in order of increasing chl *a* concentration (Table 3).

Of all cyanobacterial taxa, including those not identified, *Synechococcus* spp. were most abundant in terms of 16S rDNA copy number in the majority of the water bodies (Fig 3A). Of the three identified cyanobacterial taxa, *Synechococcus* spp. were predominant in terms of biovolume as well, despite their being considerably smaller than *M*. *aeruginosa* and *Dolichospermum* spp. (Fig 3B). The predominance of *Synechococcus* spp., however, gradually became less pronounced as the total phytoplankton abundance or trophic status of a water body increased. This was more obvious in terms of biovolume than rDNA; water bodies of higher trophic status (e.g., NU, HU, TS) were dominated by *M*. *aeruginosa* or *Dolichospermum* spp. instead of *Synechococcus* spp.

**Fig 4:**
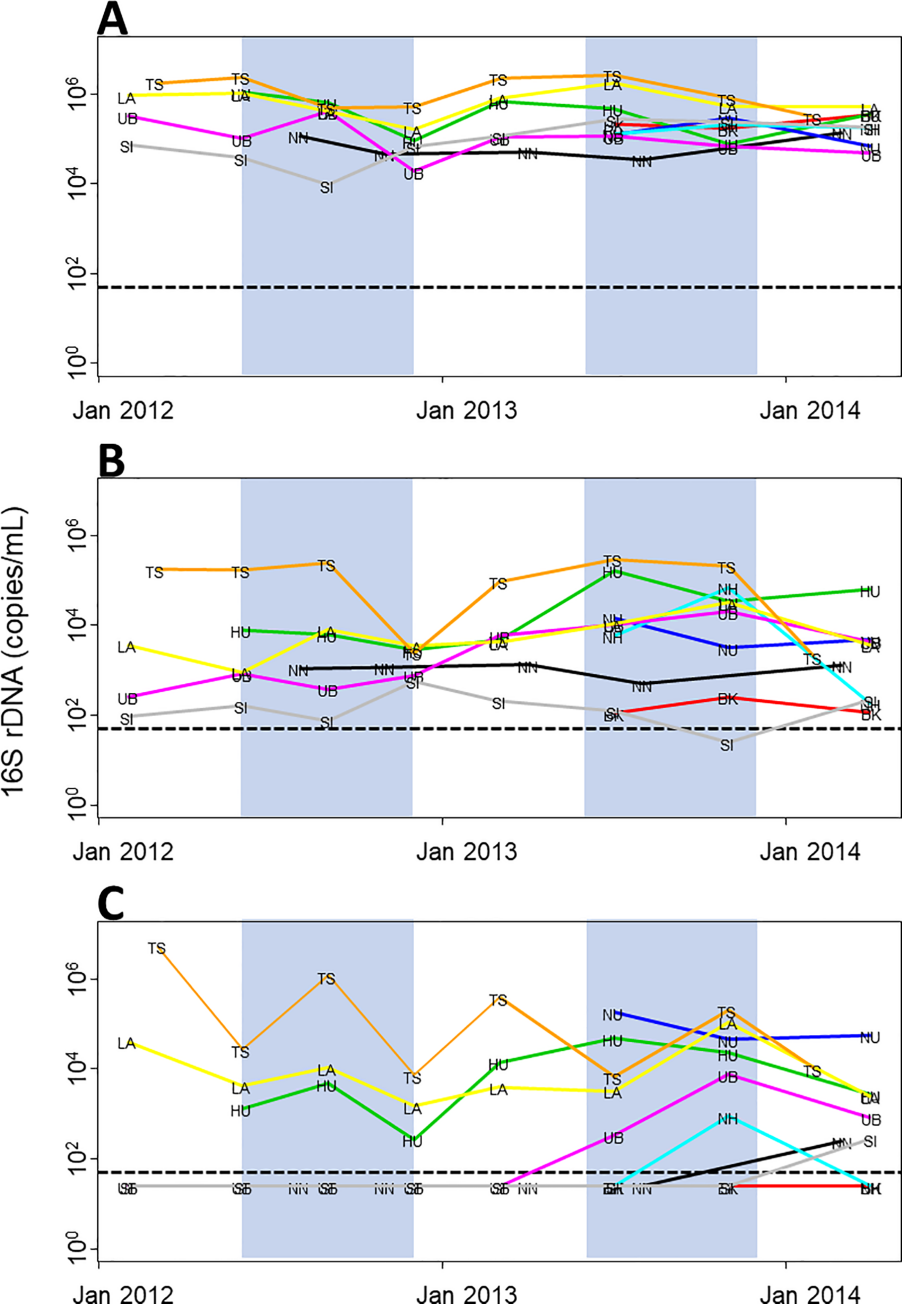
Seasonal dynamics of cyanobacteria. 16S rDNA densities during the study period for (A) *Synechococcus* spp., (B) *Microcystis aeruginosa*, and (C) *Dolichospermum* spp. Shaded areas represent the wet season (May–October) as defined in text. Dashed horizontal lines represent detection limits.

Throughout the study period, the densities of *Synechococcus* spp. remained high and relatively constant (1.0 × 10^4^ to 1.0 × 10^6^ copies/mL) (Fig 4). *Microcystis aeruginosa* was ubiquitous, except for oligotrophic SI in Thailand where the density of both *M*. *aeruginosa* and *Dolichospermum* spp. remained low, near or below the detection limit. Despite NN in Lao PDR having the lowest phosphorus level (Table 4), *M*. *aeruginosa* persisted there at moderate densities (around 1.0 × 10^3^ copies/mL). *Dolichospermum* spp. occurred only in TS, LA, and HU in 2012; they became more widespread in the following years except in BK, where they were never found.

Higher cyanobacterial densities were generally associated with shallower and more eutrophic water bodies for all three algae (Figs 2 and 4). The highest densities were almost always recorded from eutrophic TS in Cambodia, whereas the lowest were either from oligotrophic NN or SI. During our field sampling, the only, but quite conspicuous, bloom occurred in TS near Siem Reap, a famous tourist destination, when *Dolichospermum* spp. recorded their maximum density (5.2 × 10^6^ copies/mL) in February 2012.

The density of *Synechococcus* spp. varied synchronously among water bodies throughout the study period (*W* = 0.457, *P* = 0.019), although the variability did not correspond well with the annual cycle of dry and wet seasons (Fig 4). *A posteriori* tests revealed that there was significant synchrony in algal densities between LA and the other water bodies (*W*_*j*_ = 0.643, *P* = 0.003) and also between TS and the others (*W*_*j*_ = 0.586, *P* = 0.010). No synchrony was observed for *M*. *aeruginosa* (*W* = 0.240, *P* = 0.301). A limited, insignificant synchrony appeared to exist for *Dolichospermum* spp. (*W* = 0.217, *P* = 0.055).

**Fig 5:**
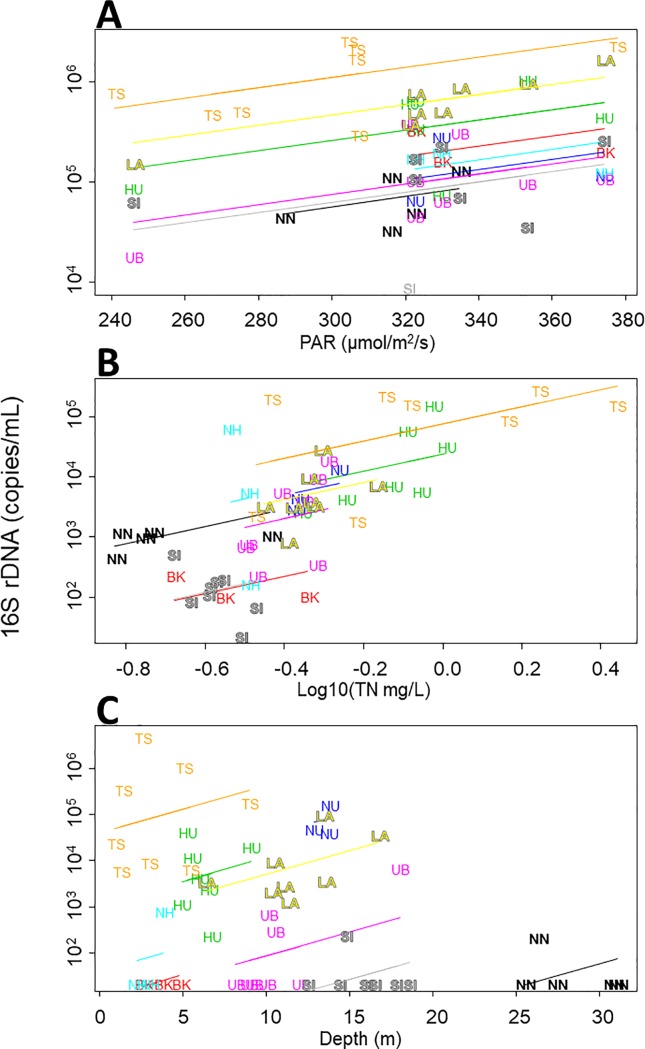
Regression models for the three algal taxa. 16S rDNA densities of (A) *Synechococcus* spp., (B) *Microcystis aeruginosa*, and (C) *Dolichospermum* spp. plotted against a respective significant predictor, with separate regression lines for different water bodies, constituting another significant predictor, water. PAR, photosynthetically active radiation; TN, total nitrogen.

Multiple linear regression included the categorical variable water in all three models of algal densities (Table 5, Fig 5). PAR was also selected as a significant predictor for the *Synechococcus* model (*R*^2^ = 0.997, F_10,43_ = 1360, *P* < 0.001). The positive influence of PAR was recognizable only within water bodies; no positive or negative overall relationship existed between PAR and the algal density when all data were pooled across the water bodies (Fig 5A). In the *Microcystis* model (*R*^2^ = 0.977, F_10,43_ = 186.1, *P* < 0.001), TN was a significant predictor in addition to water, such that *Microcystis* was more abundant in seasons when TN was high within each water body, and in water bodies with higher background TN levels as well (Fig 5B). Depth was selected as a significant predictor along with water in the *Dolichospermum* model (*R*^2^ = 0.962, F_10,43_ = 109.2, *P* < 0.001). Interestingly, the sign of the depth variable was positive (0.102), although the overall relationship between water depth and algal density was significantly negative (*r* = –0.356, *P* = 0.009). The algae were more abundant when the water level was higher (i.e., deeper) in each water body, but they actually were more dominant in shallower water bodies (Fig 5C).

**Table 5:** Estimates of coefficients included in multiple regression models, and their significance, for *Synechococcus* spp., *Microcystis aeruginosa*, and *Dolichospermum* spp.

Taxon	Variable	Coefficient	SE	*t*-value	*P*
*Synechococcus* spp.	PAR	0.005	0.001	3.583	0.001
	Water TS	4.507	0.441	10.218	<0.001
	Water LA	4.138	0.479	8.644	<0.001
	Water HU	3.885	0.479	8.110	<0.001
	Water BK	3.620	0.525	6.900	<0.001
	Water NH	3.486	0.525	6.644	<0.001
	Water NU	3.388	0.525	6.457	<0.001
	Water UB	3.341	0.479	6.980	<0.001
	Water SI	3.262	0.479	6.815	<0.001
	Water NN	3.219	0.474	6.797	<0.001
*Microcystis aeruginosa*	TN	1.423	0.570	2.498	0.016
	Water TS	4.882	0.218	22.430	<0.001
	Water HU	4.379	0.241	18.202	<0.001
	Water NH	4.348	0.453	9.589	<0.001
	Water NU	4.261	0.398	10.697	<0.001
	Water LA	4.195	0.288	14.549	<0.001
	Water NN	4.025	0.489	8.235	<0.001
	Water UB	3.881	0.310	12.512	<0.001
	Water SI	2.943	0.392	7.516	<0.001
	Water BK	2.919	0.461	6.339	<0.001
*Dolichospermum* spp.	Depth	0.102	0.045	2.248	0.030
	Water TS	4.615	0.301	15.313	<0.001
	Water NU	3.523	0.738	4.776	<0.001
	Water HU	3.053	0.394	7.757	<0.001
	Water LA	2.694	0.584	4.611	<0.001
	Water NH	1.611	0.439	3.674	0.001
	Water BK	1.024	0.449	2.280	0.028
	Water UB	0.933	0.554	1.684	0.100
	Water SI	–0.079	0.760	–0.104	0.917
	Water NN	–1.273	1.316	–0.967	0.339

Coefficients for water are listed in decreasing order within each taxon.

## Discussion

### Dynamics of the picoplankton *Synechococcus* spp

The picocyanobacteria *Synechococcus* spp. were ubiquitous among the large water bodies of the Mekong River Basin. It has been widely accepted that *Synechococcus* spp. and picophytoplankton in general are common in large, deep oligo–mesotrophic water bodies because of their very rapid nutrient uptake rates and low-light adaptation [5,6,39], and that their contribution to overall algal biomass declines with increasing trophic status [40]. Although our observations of the percent contributions of cyanobacteria conform to this model (Fig 3), actual algal densities of not only bloom-forming cyanobacteria but also of *Synechococcus* spp. were higher in nutrient-rich, eutrophic waters (Fig 4) than in oligotrophic waters, with the maximum densities typically recorded in the shallow, turbid TS. Turbid waters often favor picophytoplankton regardless of trophic status because picophytoplankton have a competitive advantage over large phytoplankton under turbid or low-light conditions and because the inorganic components partially responsible for the turbidity have a significant negative effect on grazing [9].

The synchronous pattern of *Synechococcus* spp. densities across water bodies was remarkable despite the fact that the water bodies are diverse in terms of origin, size, and trophic status, and that some waters are separated by more than 500 km. The synchrony may be explained by the strong influence of PAR on the algal dynamics as revealed by regression analysis. Values for solar irradiance, which parallels PAR, measured at multiple university campuses in northeastern Thailand, where most of the water bodies in this study are located, were fairly well correlated with each other (*r* > 0.8) (S1 Fig). Therefore, light conditions for photosynthetic activity were likely sufficiently uniform over the vast area of the Lower Mekong Basin to allow *Synechococcus* spp. to vary synchronously among the water bodies.

Some water bodies in the Mekong Basin are well known for high commercial catches of freshwater herring (*Clupeichthys aesarnensis*), a lucrative fishery in the region. This species constitutes 50–60% of the catch in NN and about one-third in SI [41]. Whereas the fish themselves feed mainly on planktonic crustaceans [42], the crustaceans in turn feed on smaller zooplankton such as heterotrophic nanoflagellates and small ciliates that are the main consumers of photosynthetic picoplankton [13,14,43]. Even large mesozooplankton such as *Daphnia* are capable of filtering and ingesting particles as small as 0.5 μm [44], thereby directly consuming picocyanobacteria and contributing to energy transfer to higher trophic levels [10,45,46]. The ubiquitous and highly abundant *Synechococcus* spp. would serve as an important carbon source for fishes and support local fisheries and aquaculture in future reservoirs in the Mekong River Basin.

### Dynamics of two bloom-formers: *M*. *aeruginosa* and *Dolichospermum* spp

The only visible bloom that occurred during the study period was the severe bloom (5.2 × 10^6^ copies/mL) of *Dolichospermum* spp. in February 2012 in TS. The magnitude of this bloom was comparable to or greater than any ever recorded worldwide [35]. Previous studies have not reported any massive algal blooms from TS [47], although the dominance of *Anabaena* species during the dry season has been documented [48]. In TS and a few eutrophic and mesotrophic reservoirs such as HU and NU, the densities of both *M*. *aeruginosa* and *Dolichospermum* spp. occasionally approached 1.0 × 10^6^ copies/mL, above which *Microcystis* formed a thick surface bloom in a temperate eutrophic lake [37]. In fact, a bloom of *Microcystis* was observed in a reservoir in northeast Thailand when the water temperature reached its maximum of 30°C in June and July 2011 [20].

The *Microcystis* density was determined primarily by the system-specific variable of water and secondarily by TN. However, because there were correlations between some of the nutrient concentrations (i.e., multicollinearity), the seasonal dynamics of *M*. *aeruginosa* could have been almost equally explained, for example, by TP (*r* = 0.873, *P* < 0.001). Although it is plausible that *M*. *aeruginosa*, a non-N_2_-fixing cyanobacterium, is indeed limited by nitrogen in the Mekong lakes and reservoirs, this cannot be confirmed through the correlative analysis of nutrients and the alga. Nevertheless, TN can be considered a useful metric for predicting the abundance of *M*. *aeruginosa* in these waters, as also demonstrated in numerous other water bodies around the globe [49,50]. Whether *Microcystis* was nitrogen- or phosphorus-limited, the factor(s) controlling its dynamics was highly specific to individual water bodies as its densities were not at all synchronized across water bodies, unlike *Synechococcus* spp.

*Dolichospermum* spp. occurred only in shallow waters, but not in all shallow waters, because they were never found in BK and rarely found in NH, both of which are shallow and both of which were originally natural lakes or swamps. Note that BK had very low *Microcystis* densities as well. These waters have well-preserved aquatic macrophyte communities in both littoral and pelagic ecosystems from before they were dammed for irrigation [31]; this presumably has suppressed the growth of the bloom-forming cyanobacteria through competition for nutrients or light [51].

**Fig 6:**
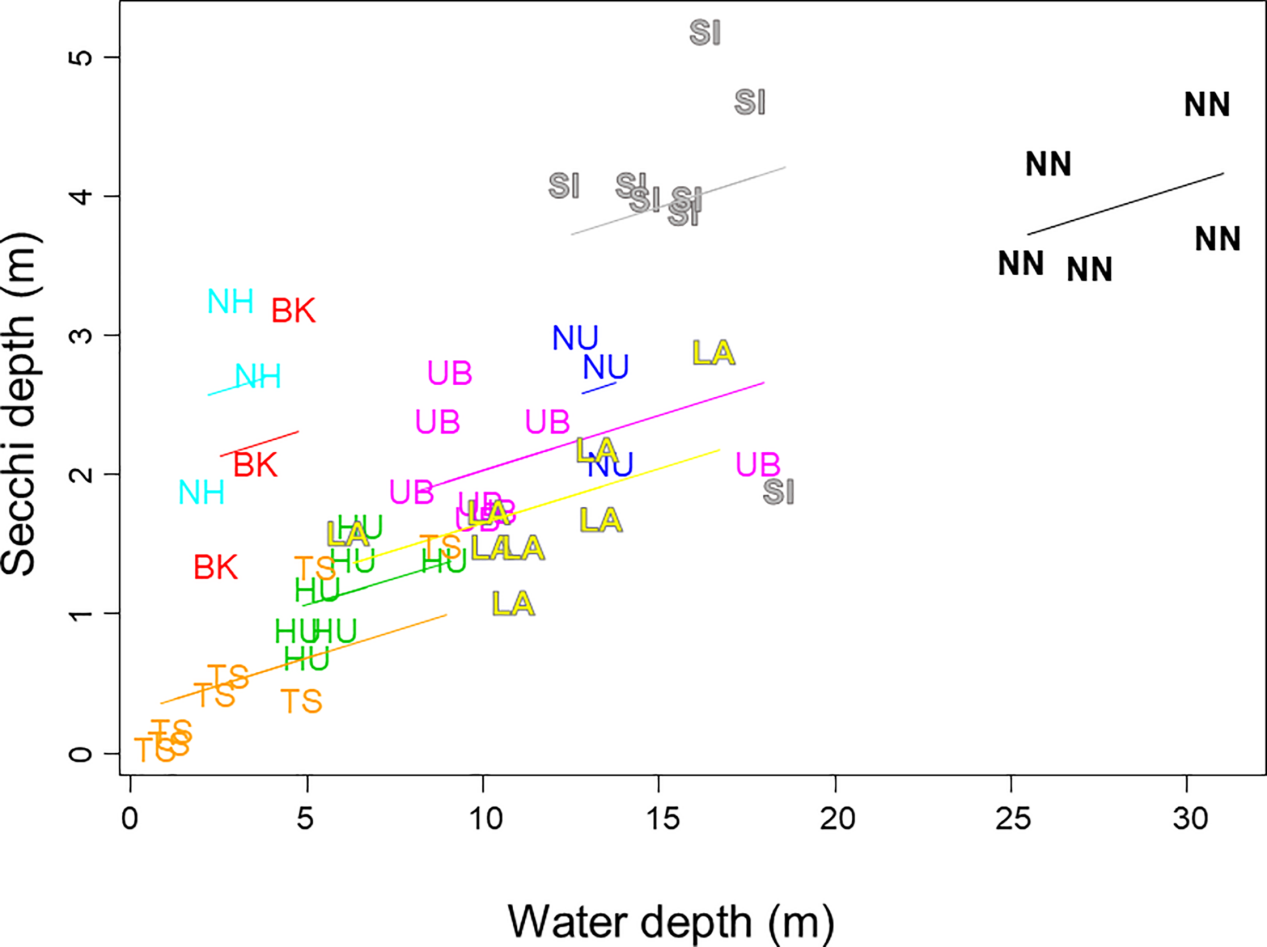
Secchi depth plotted against its significant predictor, water depth. Separate regression lines are shown for each water body to represent another significant predictor, water.

The seasonal dynamics of *Dolichospermum* was influenced by changes in water level after the water effect was accounted for, such that the higher the water level, the greater the algal density. Like solar irradiance, water level can vary concordantly among water bodies over a wide geographical range in response to rainfall and other climatological or hydrological events [31,52]. As a result, there was also a weak synchrony in *Dolichospermum* densities throughout the Mekong River Basin. The positive relationship between water level and the algal density may seem contradictory to the aforementioned propensity of this alga to occur in shallower waters. The increased water level of lakes and reservoirs results from extreme rainfall events that are common during the rainy season in the Mekong Basin, draining waste materials and fertilizers from uplands as non-point-source pollution and seasonally enriching the waters with nutrients [53]. However, our preliminary analysis refuted this nutrient enrichment hypothesis for the positive water level effect on *Dolichospermum* spp. abundance because concentrations of nitrogen and phosphorus were either negatively correlated with or had no correlations with water level. Instead, the water level was positively correlated with Secchi depth, such that the transparency increased in deeper water bodies, and in seasons with higher water levels in a given water body (*R*^2^ = 0.957, F_10,43_ = 96.45, *P* < 0.001; Fig 6). Therefore, the increased densities of *Dolichospermum* spp. with increasing water level may actually be a response to increased transparency and an associated increase in light penetration.

### Conclusions

This study predicts that the picocyanobacteria *Synechococcus* spp. will likely be predominant or even ubiquitous in newly created reservoirs in the Mekong River Basin. However, because their productivity is determined primarily by light conditions (i.e., PAR) rather than by nutrients or other controlling factors, it is technically not feasible to enhance algal production in the hope of increasing fish production. Of the two bloom-forming cyanobacteria, *M*. *aeruginosa* density increased in response to increased nutrient levels, especially nitrogen. The other bloom former *Dolichospermum* spp. dominated in shallow waters and proliferated as water level increased, possibly due to an associated improvement in light conditions.

The most effective and promising management strategy for preventing or reducing the frequency of harmful algal blooms in future reservoirs in the Mekong River Basin would be to limit anthropogenic inputs of nutrients. Because aquatic macrophytes appear to play an important role in suppressing algal blooms, especially those of *Dolichospermum* spp., restoration or creation of littoral and pelagic ecosystems dominated by aquatic macrophytes could be an effective, long-term strategy for sustainable reservoir management in this region.

With the number of dams and reservoirs increasing in the future, whole-system primary production will most likely increase in the Mekong River. However, further research is required to determine whether this translates into increased whole-system fisheries production and therefore higher food security for the Mekong riparian countries.

## Supporting information

S1 Fig Solar irradiance data measured during the study period at 4 locations throughout the study area of the Mekong basin. (Courtesy of Solar Energy Research Laboratory, Silapakorn University).
(TIF)
