# Supplementary figures and images for: The dynamics of pico-sized and bloom-forming cyanobacteria in large water bodies in the Mekong River Basin
Source: PLoS One. 2017 Dec 22;12(12):e0189609. doi: 10.1371/journal.pone.0189609 (PMC5741221; doi:10.1371/journal.pone.0189609)

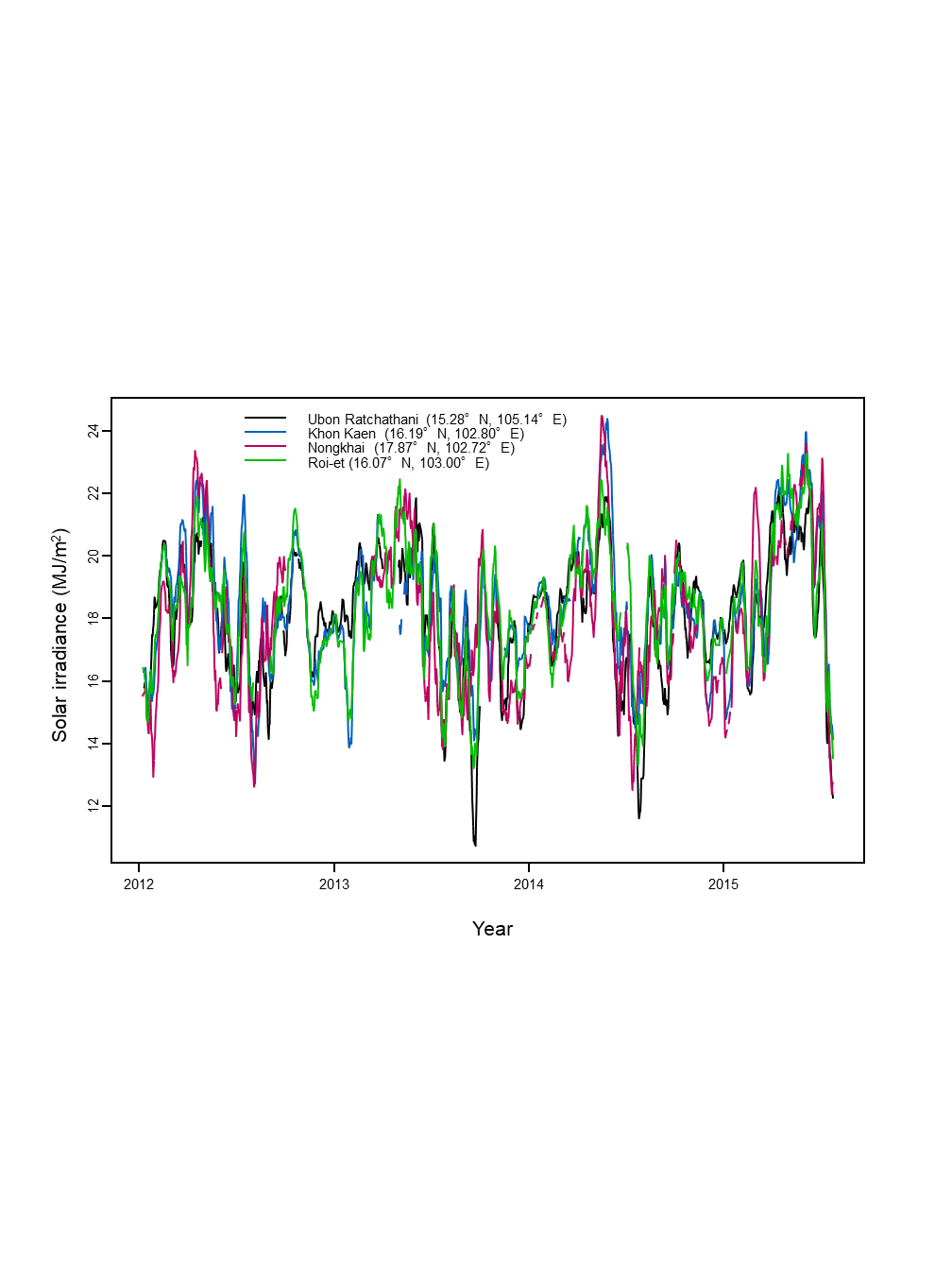

Supplement: S1 Fig — (Courtesy of Solar Energy Research Laboratory, Silapakorn University). (TIF) [file pone.0189609.s001.tif]
